# Machine learning models based on quantitative dynamic contrast-enhanced MRI parameters assess the expression levels of CD3^+^, CD4^+^, and CD8^+^ tumor-infiltrating lymphocytes in advanced gastric carcinoma

**DOI:** 10.3389/fonc.2024.1365550

**Published:** 2024-03-14

**Authors:** Huizhen Huang, Zhiheng Li, Dandan Wang, Ye Yang, Hongyan Jin, Zengxin Lu

**Affiliations:** ^1^ Department of Radiology, Shaoxing People’s Hospital, Shaoxing Hospital, Zhejiang University School of Medicine, Shaoxing, China; ^2^ Department of Pathology, Shaoxing People’s Hospital, Shaoxing Hospital, Zhejiang University School of Medicine, Shaoxing, China

**Keywords:** dynamic contrast-enhanced magnetic resonance imaging, advanced gastric carcinoma, machine learning, CD3^+^, CD4^+^, CD8^+^

## Abstract

**Objective:**

To explore the effectiveness of machine learning classifiers based on dynamic contrast-enhanced magnetic resonance imaging (DCE-MRI) in predicting the expression levels of CD3^+^, CD4^+,^ and CD8^+^ tumor-infiltrating lymphocytes (TILs) in patients with advanced gastric cancer (AGC).

**Methods:**

This study investigated 103 patients with confirmed AGC through DCE-MRI and immunohistochemical staining. Immunohistochemical staining was used to evaluate CD3^+^, CD4^+^, and CD8^+^ T-cell expression. Utilizing Omni Kinetics software, radiomics features (K^trans^, K_ep_, and V_e_) were extracted and underwent selection via variance threshold, SelectKBest, and LASSO methods. Logistic regression (LR), support vector machine (SVM), random forest (RF), and eXtreme Gradient Boosting (XGBoost) are the four classifiers used to build four machine learning (ML) models, and their performance was evaluated using 10-fold cross-validation. The model’s performance was evaluated and compared using the area under the receiver operating characteristic curve (AUC), accuracy, sensitivity, specificity, positive predictive value, and negative predictive value.

**Results:**

In terms of CD3^+^, CD4^+^, and CD8^+^ T lymphocyte prediction models, the random forest model outperformed the other classifier models in terms of CD4^+^ and CD8^+^ T cell prediction, with AUCs of 0.913 and 0.970 on the training set and 0.904 and 0.908 on the validation set, respectively. In terms of CD3^+^ T cell prediction, the logistic regression model fared the best, with AUCs on the training and validation sets of 0.872 and 0.817, respectively.

**Conclusion:**

Machine learning classifiers based on DCE-MRI have the potential to accurately predict CD3^+^, CD4^+^, and CD8^+^ tumor-infiltrating lymphocyte expression levels in patients with AGC.

## 1 Introduction

Although incidence and mortality have decreased in recent years, gastric cancer remains the fifth most common disease and the fourth leading cause of cancer death worldwide (1). The most common form of treatment for stomach cancer is still traditional surgical resection (2). Although only approximately 30% of stomach cancer patients are thought to be suitable candidates for radical resection, the alarming truth is that the great majority of patients receive a diagnosis when the disease has already progressed (3).

A major resurgence of hope has emerged on the horizon of advanced gastric cancer (AGC) treatment in recent years, ushered in by new immunotherapy research (4, 5). The use of immunosuppressants targeting programmed cell death ligand 1 (PD-L1) and/or programmed cell death 1 (PD-1) in particular heralds an entirely new age of immunotherapy in cancer treatment (6). Immunotherapy, when paired with other treatments, has significantly boosted the survival rate of patients with gastric cancer (7). The level of T lymphocyte infiltration in the tumor microenvironment is crucial for tumor immunotherapy success (8, 9). T lymphocytes are classified into several functional subsets, including other subtypes, such as helper (CD3^+^CD4^+^) T cells and killer (CD3^+^CD8^+^) T cells. The majority of T lymphocytes exhibit CD3, which is known as a biomarker for T lymphocytes with antitumor activity and is a significant prognostic indicator for overall survival and recurrence (10). The majority of antitumor effector cells are CD8^+^ T cells, and it has been established that CD8^+^ tumor-infiltrating lymphocytes (TILs) are crucial in anti-PD-1/PD-L1 therapy. A key component and predictor of the prognosis for gastric cancer is thought to be CD8^+^ TILs (11). The bulk of CD4^+^ T cells are helper T lymphocytes, which are crucial for tumor surveillance because they support CD8^+^ T-cell activation and proliferation as well as collaborate on antitumor actions (12). It is possible to more correctly forecast the trajectory of tumor development and the prognosis of patients by determining the presence of CD3^+^, CD4^+^, and CD8^+^ T cells in the tumor lesion area (13). Tissue samples are now needed to assess CD3^+^, CD4^+^, and CD8^+^ T-cell infiltration in malignant tumors, but acquiring these samples requires intrusive procedures such as surgical or puncture biopsies, which limits the capacity to provide a dynamic and comprehensive assessment of infiltration. Additionally, due to the heterogeneity of the malignancy, local samples are frequently not entirely typical of the whole tumor. Therefore, a noninvasive, repeatable approach to evaluate the infiltration of CD3^+^, CD4^+^, and CD8^+^ T cells in malignancies is urgently needed in clinical settings.

Radiomics is a rapidly expanding field that has shown significant promise in recent years. It shows enormous potential in a number of areas, such as disease diagnosis, tumor staging, protein expression detection, and prognosis prediction (14, 15). Radiomics has been shown to have considerable benefits in the treatment of stomach cancer (16). Recent research has shown that combining dynamic contrast-enhanced MRI (DCE-MRI) with radiomics analysis can produce promising findings in analyzing protein expression (17). On the one hand, radiomics can rapidly extract quantitative features from medical images, providing useful information for auxiliary diagnosis. DCE-MRI, on the other hand, not only provides deeper insight into blood vessel development and perfusion than other imaging techniques but also has superior spatial resolution and interobserver agreement of results (18).

The goal of this study was to determine whether the DCE-MRI-based noninvasive prediction model could predict the infiltration of CD3, CD4, and CD8 T-cell expression levels in advanced gastric cancer. Our findings could help in identifying patients who respond well to immunotherapy.

## 2 Materials and methods

### 2.1 Patients

The ethics review boards of our hospitals granted their approval for this retrospective research, and the patient’s informed consent was not needed.

**Figure 1:**
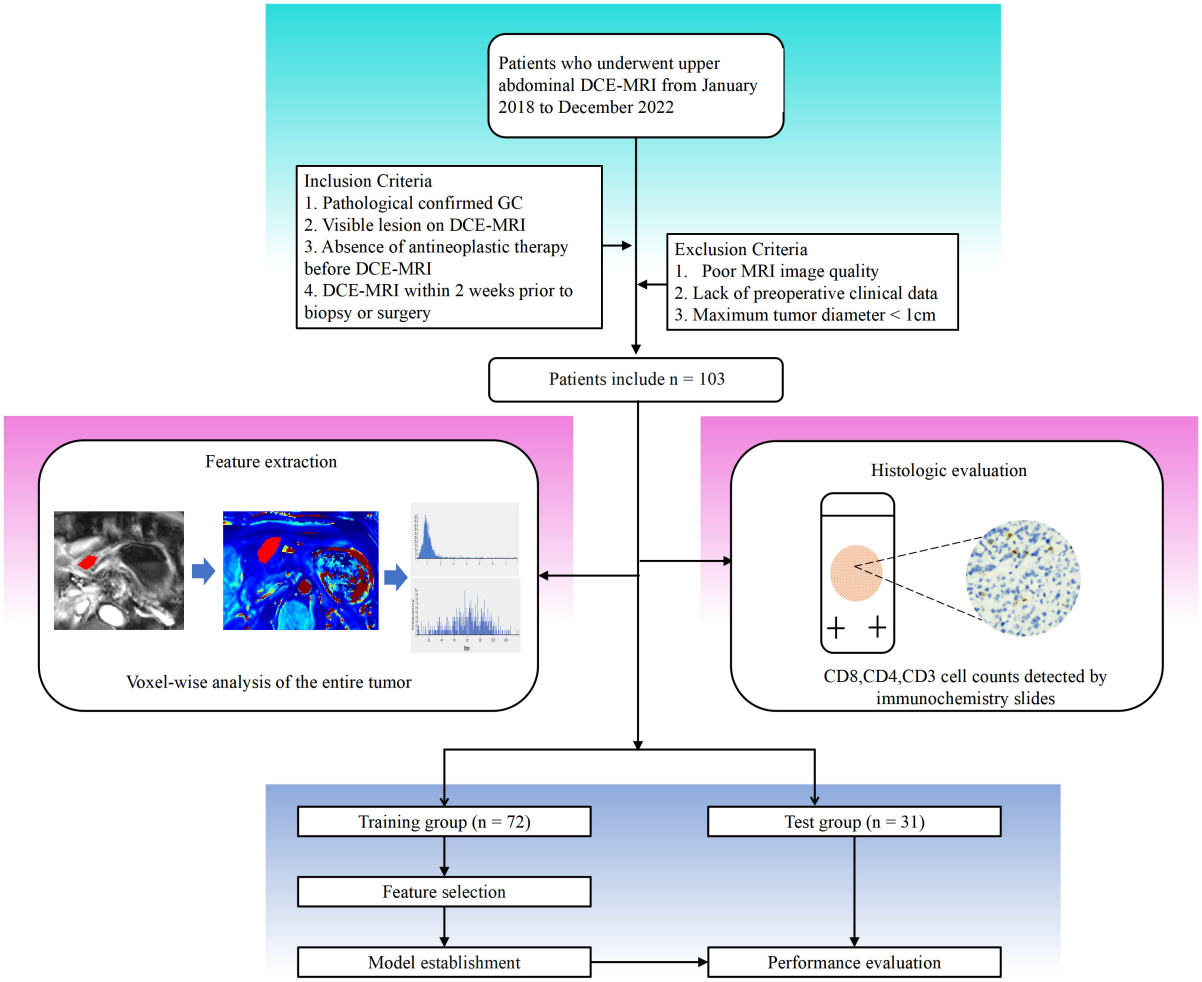
Workflow of this study. Detailed information on inclusion and exclusion of study subjects. Imaging histologic analysis and histologic assessment were performed separately. Feature screening was performed to construct the imaging histology assessment model.

Between April 2018 and July 2022, data were collected from patients pathologically confirmed with AGC via biopsy or surgery in our hospital. The specific inclusion criteria were as follows: (1) AGC established histologically; (2) visible lesion on DCE-MRI; (3) no anticancer therapy before DCE-MRI; and (4) DCE-MRI within two weeks of biopsy or surgery. These exclusion criteria were as follows: (1) significant imaging abnormalities that hampered lesion characterization; (2) maximum tumor diameter of 1 cm; and (3) absence of preoperative clinical data. Ultimately, 103 people were enrolled in the research study (**Figure 1**).

### 2.2 MRI scanning

Before the MRI, all patients received the following preparation: (1) fasted for 8 hours to allow the gastrointestinal tract to empty. (2) To suppress gastrointestinal motility, 10 mg anisodamine (Hangzhou Minsheng Pharmaceutical Co., LTD., China) was administered intramuscularly 10 minutes before the examination if there were no contraindications (e.g., glaucoma, asthma, or serious heart disease). (3) Patients were given 800-1000 mL of warm water orally 5 minutes before the exam to expand the stomach cavity.

For the MRI studies, a typical 12-channel phased-array body coil was employed in conjunction with a 3.0T MRI scanner (Verio, Siemens, Germany). The patient was lying supine during the examination, and the entire stomach was covered by the scanning field. Following a standard plain scan (T1-weighted image, T2-weighted image), a DCE-MRI scan was needed for all patients. Free-breathing is employed during DCE-MRI scans, which are performed utilizing a three-dimensional, radial volumetric interpolated, breath-hold assessment approach. Initially, the following parameters were utilized for multiangle cross-sectional T1WI in the axial plane scan: repeat time: 3.25 ms; echo time: 1.17 ms; FOV: 350 × 284 mm; matrix: 288 × 164; layer thickness: 5 mm; scan at various flip angles (5°, 10°, and 15°) for 6.5 s each, for a total of 19.5 s. The next step employed multiphase dynamic enhanced scanning with the following parameters: the Flip angle was set to 10°, 35 phases were scanned, and the total scanning time was 227.5 s. All other parameters were left at their previous values. In phase 3, a gadolinium contrast material (Omniscan, GE Healthcare, China) was injected through the median elbow vein using a high-pressure injector. The injection dose and rate were set at 0.1 mmol/kg and 3.5 ml/s, respectively. To flush the region, 20 ml of saline was administered at the same flow rate.

### 2.3 Immunohistochemical staining and analysis

**Figure 2:**
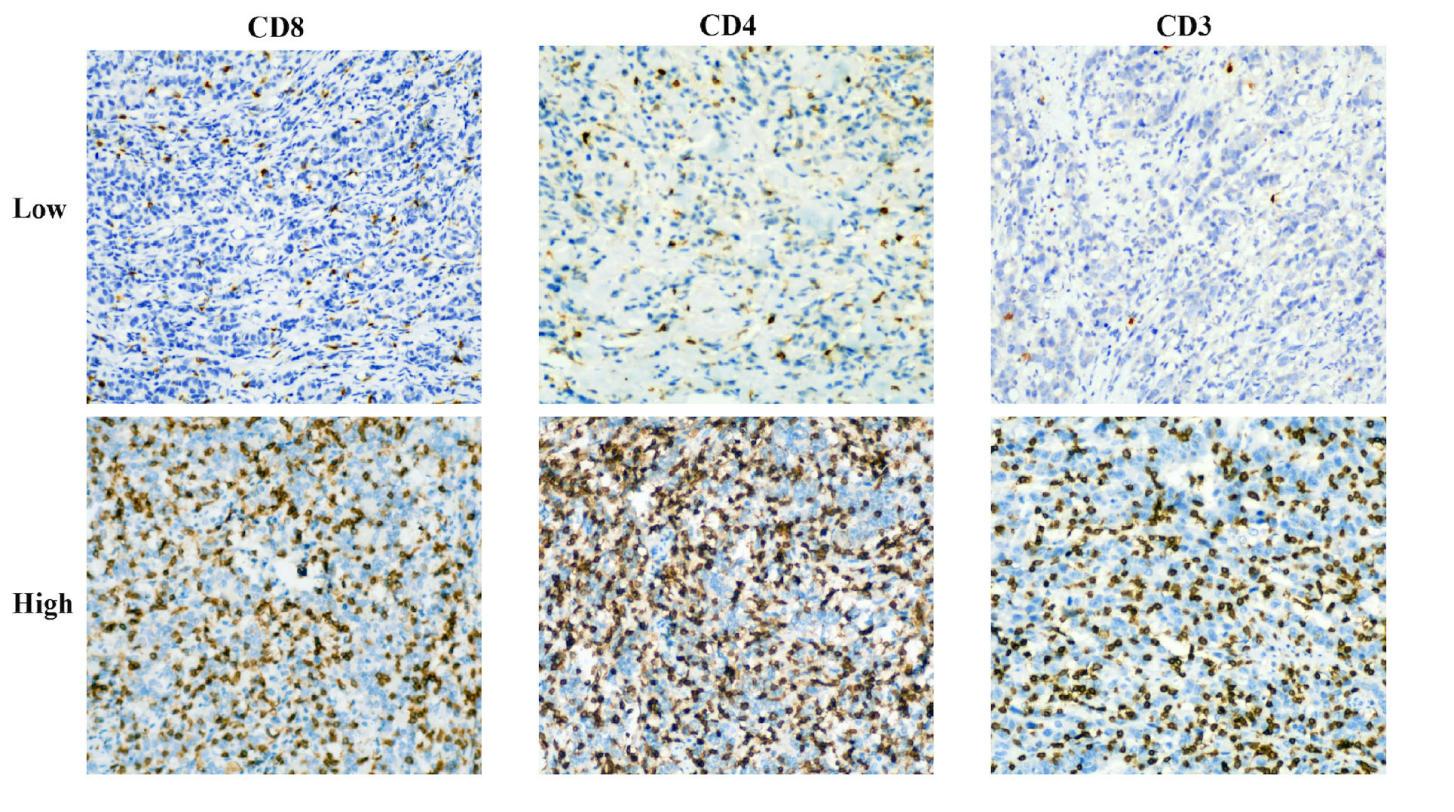
Representative immunohistochemical staining images of CD3, CD4, and CD8 cells in patients with advanced gastric cancer.

The expression of CD3^+^, CD4^+^, and CD8^+^ T cells in gastric cancer tissues was examined using immunohistochemistry (IHC). Pathological samples for gastric cancer were obtained through gastroscopic biopsy or surgery. All GC tissues that had been formalin-fixed and paraffin-coated were sliced into 4-μm-thick slices. Immunohistochemical staining was carried out using mouse anti-CD8 monoclonal antibody (1:200, GT211202, Gene Tech, Shanghai, China), rabbit anti-CD4 monoclonal antibody (1:200, GT219102, Gene Tech, Shanghai, China), or rabbit anti-CD3 monoclonal antibody (1:200, GT219001, Gene Tech, Shanghai, China). Overnight, the portions were kept in a 4°C refrigerator. The samples were then stained with a secondary antibody (K5009, Dako, Beijing, China) and incubated at 37°C for 10 min. Hematoxylin was employed as a counterstain, and diaminobenzidine (DAB) was utilized to designate the antibody. Before being examined under a microscope, sections were made transparent, dried, and mounted. Two knowledgeable pathologists conducted a double-blind examination of the immunohistochemical results. A low-power microscope was used to examine the complete tissue field before five randomly chosen fields were examined using a high-power (X40) microscope (**Figure 2**). The tumor tissue and stroma surrounding it, as well as cancer cell nests, were all included in the counting field. Patients were divided into two groups based on the median after CD3^+^, CD4^+^, and CD8^+^ T-cell expression was evaluated based on the average number of positively stained cells, according to an earlier study (19).

### 2.4 Image data analysis and processing

We used Omni Kinetics (GE Healthcare, China) software to postprocess the DCE-MRI image data of all qualified AGC patients.

**Figure 3:**
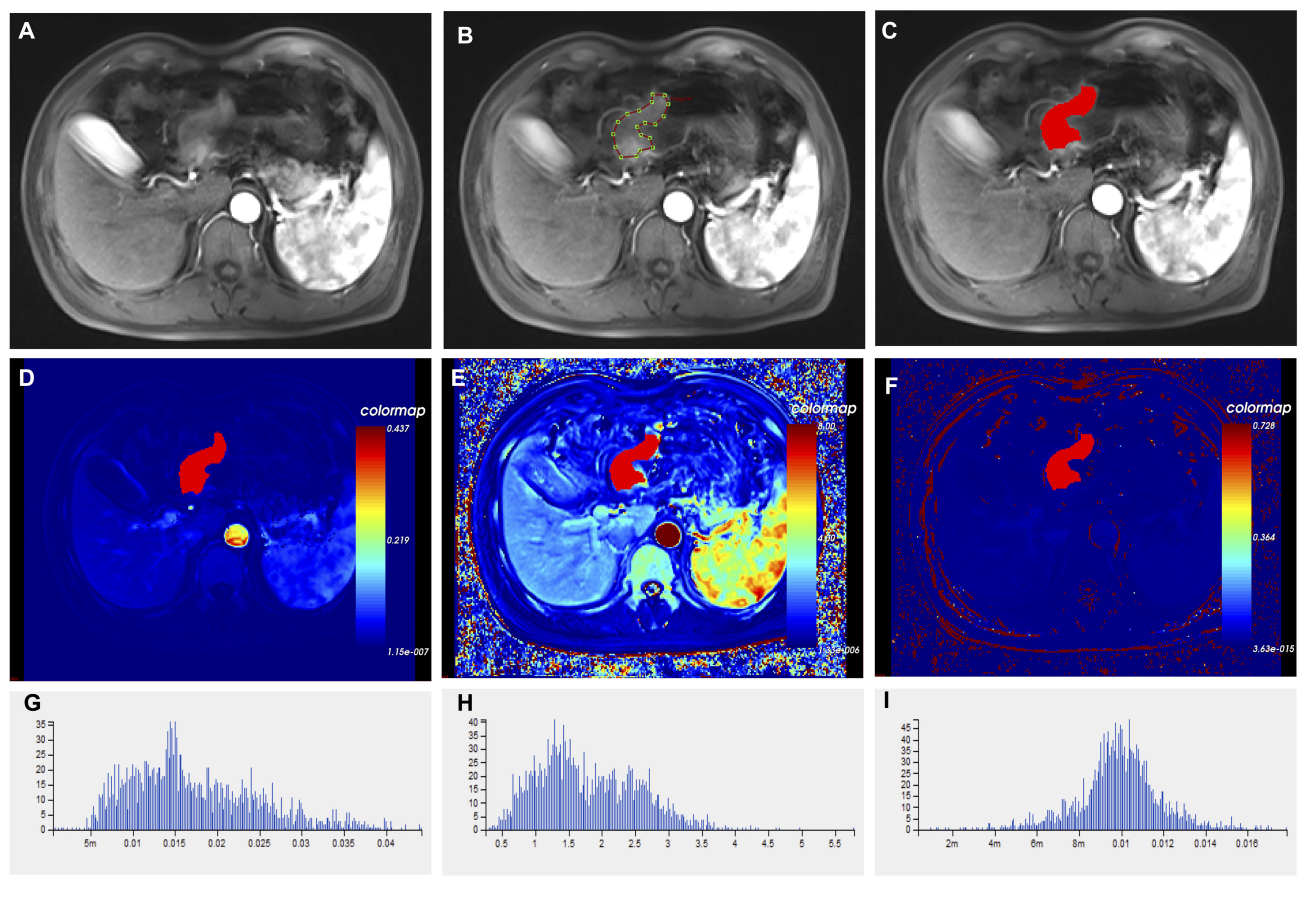
Histograms of different imaging modalities and quantitative perfusion parameters in patients with advanced gastric cancer. **(A)** Axial T1-weighted images showed a mass with an irregular and thickened gastric wall. **(B)** ROIs were placed manually in axial T1-weighted images. **(C)** Outlining the target area for eventual fusion into a three-dimensional structure. **(D)** Volume transfer constant (K^trans^) plot of the ROI. **(E)** The plot of the reverse reflux rate constant (K_ep_) for the ROI. **(F)** The plot of extracellular extravascular volume fraction (V_e_) of ROI. **(G)** Histogram of K^trans^ values. **(H)** Histogram of K_ep_ values. **(I)** Histogram of V_e_ values. ROI, Region of interest.

Regions of interest (ROI) labeling: T1-mapping multi-flip Angle (5°, 10°, and 15°) sequence and dynamic enhancement sequence scan images were imported into the OK software workstation for post-processing. A variable flip Angle method was used to convert the signal intensity to the omnipowerful scanning concentration, and the cross-section was used as the main measurement plane. The abdominal aorta was manually selected to obtain the artery input function type (AIF Type) for image post-processing. A nonlinear registration framework (free deformation algorithm) was used to correct artifacts due to body motion (e.g., breathing) between consecutive DCE-MRI scans. The hemodynamic model Tofts model was selected to calculate the pharmacokinetic perfusion parameters. The lesion was delineated in 3-5 layers, avoiding necrotic and healthy gastric tissue, and the lesion was integrated into a 3D-ROI for quantitative analysis and calculation (**Figure 3**). Two experienced radiologists (radiologist 1 with 5 years experience and radiologist 2 with 8 years experience), who were unaware of the clinical and pathological data of the patients, segmented the measurements and averaged three times.

Feature extraction: the pharmacokinetic parameters of the whole tumor were generated, and the Tofts model was used to calculate the pharmacokinetic parameters, including the transfer rate constant from plasma to extravascular extracellular space (Ktrans), the transfer rate constant from extravascular extracellular space back to plasma (Kep) and the volume fraction of extravascular extracellular space (Ve). The software then automatically extracted the pharmacokinetic parameter features of the whole tumor from the three perfusion maps, a total of 201 features. These features included five categories: first order, histogram, gray level co-occurrence matrix, Haralick, and run-length matrix. The specific operation interface of the Omni Kinetics software is shown in **Supplementary Material**.

### 2.5 Interobserver variability evaluation

30 patients were recruited at random to assess the consistency of radiomics feature extraction by various observers. Intraclass correlation coefficients (ICCs) were calculated for tumor segmentation performed separately by readers 1 and 2, one week apart. Intra-group consistency analysis was then done on the features outlined by reader 1, followed by inter-group consistency analysis on the same 30 patients’ features delineated by readers 1 and 2. The reproducibility of radiomics characteristics retrieved from DCE-MRI was rated satisfactory, with both intraobserver and interobserver ICC values more than 0.75. These features, which showed good repeatability, were collected for further radiomics study.

### 2.6 Feature selection

The average value of each extracted radiomics feature was subtracted, its standard deviation was divided by it (a process known as Z score normalization), and all of the original feature values were then transformed into feature values with a 0-1 normal distribution. All features that are extracted may not apply to a particular activity. Therefore, a critical step for achieving the most effective result is to screen out particular features that are most pertinent to this study. In this work, three strategies for dimension reduction were used to eliminate redundant features: the variance threshold, the single variable selection method, and the least absolute shrinkage and selection operator (LASSO) method. Features with less than 0.8 variance are first eliminated by the variance cutoff. A p-value is used to assess the link between features and classification outcomes in the SelectKBest method. The screening of all characteristics with a p-value less than 0.05 is possible using this univariate feature selection technique. L1 regularization is used in LASSO regression as the cost function, with a maximum of 1000 iterations, to eliminate weakly correlated features and ultimately produce the best feature selection.

### 2.7 Construction and validation of radiomics models

Because only 103 patients were enrolled, it was impossible to evaluate the robustness of our model using the conventional method of splitting the sample into training and validation groups. Using 10-fold cross-validation, our study evaluated the resilience of the prediction model. The training data were subjected to a 10-fold internal cross-validation. The training data were divided into ten subsets; one subset was used for validation, while the other nine subsets were used for training. The next 10 iterations followed. These data were used to train different classifier models, mainly including Logistic Regression(LR); Support Vector Machine (SVM); RandomForest (RF); and eXtreme Gradient Boosting(XGBoost). The accuracy, sensitivity, specificity, positive predictive value, negative predictive value, and AUC of each classifier model in the training and test populations were calculated to assess prediction performance.

### 2.8 Statistical analyses

For statistical analysis and the creation of visualizations, GraphPad Prism 8.0, SPSS version 24.0, and R software version 4.0.2 (primarily packages for glmnet, pROC, RMS, and rmda) were utilized. The use of “glmnet” was made of the LASSO approach. The R software’s “calibrate” function from the “rms” package was used for calibration. Count data were compared using the chi-square test or Fisher’s exact probability test. Using the Mann−Whitney U test, continuous variables were compared between groups. Interclass correlation coefficients (ICC) were used to analyze the consistency of texture features extracted from ROI between the two observers; ICC >0.75 indicated satisfactory agreement. A bilateral statistical analysis was conducted, and a p-value of 0.05 or lower was deemed statistically significant.

## 3 Result

### 3.1 Characteristics of patients

An average age of 67.7 years (range, 33-88 years) was found among 103 people with advanced stomach cancer in this retrospective analysis, 77 men and 26 women. The training cohort and test cohorts were divided into two groups, one with high infiltration and the other with low infiltration, based on the levels of CD3, CD4, and CD8 infiltration. **Figure 3** illustrates instances of the IHC analysis of CD3, CD4, and CD8 expression. 122, 87, and 138, respectively, were the median CD3^+^, CD4^+^, and CD8^+^ TIL levels in the training group. **Tables 1**–**3** contain information about the clinical traits of AGC patients in the three cohorts who had high or low levels of infiltration (CD3, CD4, and CD8).

**Table 1:** Relationship between CD3 and clinicopathologic features in patients with advanced gastric cancer.

Characteristic	Training cohorts (n = 72)			Test cohorts (n = 31)		
	High (n=36)	Low (n=36)	P	High (n=16)	Low (n=15)	P
Age (mean ± SD)	66.694 ± 11.095	69.528 ± 10.308	0.272	70.500 ± 9.779	70.000 ± 7.941	0.881
BMI (mean ± SD)	22.510 ± 2.870	22.290 ± 3.291	0.767	23.250 ± 2.492	23.392 ± 3.626	0.903
Gender			0.800			0.583
Male	24 (66.667)	25 (69.444)		14 (87.500)	14 (93.333)	
Female	12 (33.333)	11 (30.556)		2 (12.500)	1 (6.667)	
Location			0.763			0.508
Cardia	6 (16.667)	5 (13.889)		2 (12.500)	4 (26.667)	
Body	12 (33.333)	15 (41.667)		4 (25.000)	2 (13.333)	
Antrum	18 (50.000)	16 (44.444)		10 (62.500)	9 (60.000)	
Differentiation level			0.343			0.366
High/Moderate	22 (61.111)	18 (50.000)		7 (43.750)	9 (60.000)	
Poor	14 (38.889)	18 (50.000)		9 (56.250)	6 (40.000)	
CEA level			0.422			0.594
Normal	25 (69.444)	28 (77.778)		9 (56.250)	7 (46.667)	
Elevated	11 (30.556)	8 (22.222)		7 (43.750)	8 (53.333)	
CA199 level			0.195			0.411
Normal	28 (77.778)	23 (63.889)		12 (75.000)	13 (86.667)	
Elevated	8 (22.222)	13 (36.111)		4 (25.000)	2 (13.333)	
CA125 level			0.276			0.682
Normal	25 (69.444)	29 (80.556)		13 (81.250)	13 (86.667)	
Elevated	11 (30.556)	7 (19.444)		3 (18.750)	2 (13.333)	

**Table 2:** Relationship between CD4 and clinicopathologic features in patients with advanced gastric cancer.

Characteristic	Training cohorts (n = 72)			Test cohorts (n = 31)		
	High (n=36)	Low (n=36)	P	High (n=16)	Low (n=15)	P
Age (mean ± SD)	68.361 ± 10.729	67.861 ± 10.693	0.846	67.938 ± 9.731	72.733 ± 7.836	0.156
BMI (mean ± SD)	22.334 ± 2.639	23.359 ± 3.359	0.160	21.651 ± 2.414	22.955 ± 3.771	0.282
Gender			0.795			0.122
Male	25 (69.444)	26 (72.222)		15 (93.750)	11 (73.333)	
Female	11 (30.556)	10 (27.778)		1 (6.250)	4 (26.667)	
Location			0.159			0.089
Cardia	4 (11.111)	7 (19.444)		4 (25.000)	2 (13.333)	
Body	12 (33.333)	17 (47.222)		0 (0.000)	4 (26.667)	
Antrum	20 (55.556)	12 (33.333)		12 (75.000)	9 (60.000)	
Differentiation level			0.345			0.605
High/Moderate	21 (58.333)	17 (47.222)		10 (62.500)	8 (53.333)	
Poor	15 (41.667)	19 (52.778)		6 (37.500)	7 (46.667)	
CEA level			0.195			0.833
Normal	23 (63.889)	28 (77.778)		9 (56.250)	9 (60.000)	
Elevated	13 (36.111)	8 (22.222)		7 (43.750)	6 (40.000)	
CA199 level			0.781			0.372
Normal	27 (75.000)	28 (77.778)		12 (75.000)	9 (60.000)	
Elevated	9 (25.000)	8 (22.222)		4 (25.000)	6 (40.000)	
CA125 level			1.000			0.682
Normal	27 (75.000)	27 (75.000)		13 (81.250)	13 (86.667)	
Elevated	9 (25.000)	9 (25.000)		3 (18.750)	2 (13.333)	

**Table 3:** Relationship between CD8 and clinicopathologic features in patients with advanced gastric cancer.

Characteristic	Training cohorts (n = 72)			Test cohorts (n = 31)		
	High (n=37)	Low (n=35)	P	High (n=16)	Low (n=15)	P
Age (mean ± SD)	67.378 ± 10.786	68.286 ± 9.747	0.714	68.750 ± 11.840	73.267 ± 6.942	0.224
BMI (mean ± SD)	22.863 ± 2.764	22.572 ± 3.089	0.679	22.828 ± 3.406	22.299 ± 3.615	0.688
Gender			0.892			0.916
Male	28 (75.676)	26 (74.286)		12 (75.000)	11 (73.333)	
Female	9 (24.324)	9 (25.714)		4 (25.000)	4 (26.667)	
Location			0.296			0.860
Cardia	9 (24.324)	5 (14.286)		2 (12.500)	1 (6.667)	
Body	9 (24.324)	14 (40.000)		5 (31.250)	5 (33.333)	
Antrum	19 (51.351)	16 (45.714)		9 (56.250)	9 (60.000)	
Differentiation level			0.101			0.379
High/Moderate	26 (70.270)	18 (51.429)		5 (31.250)	7 (46.667)	
Poor	11 (29.730)	17 (48.571)		11 (68.750)	8 (53.333)	
CEA level			0.804			0.106
Normal	23 (62.162)	27 (77.143)		12 (75.000)	7 (46.667)	
Elevated	14 (37.838)	8 (22.857)		4 (25.000)	8 (53.333)	
CA199 level			0.345			0.916
Normal	29 (78.378)	24 (68.571)		12 (75.000)	11 (73.333)	
Elevated	8 (21.622)	11 (31.429)		4 (25.000)	4 (26.667)	
CA125 level			0.191			0.779
Normal	32 (86.486)	26 (74.286)		11 (68.750)	11 (73.333)	
Elevated	5 (13.514)	9 (25.714)		5 (31.250)	4 (26.667)	

### 3.2 Radiomics analysis

**Figure 4:**
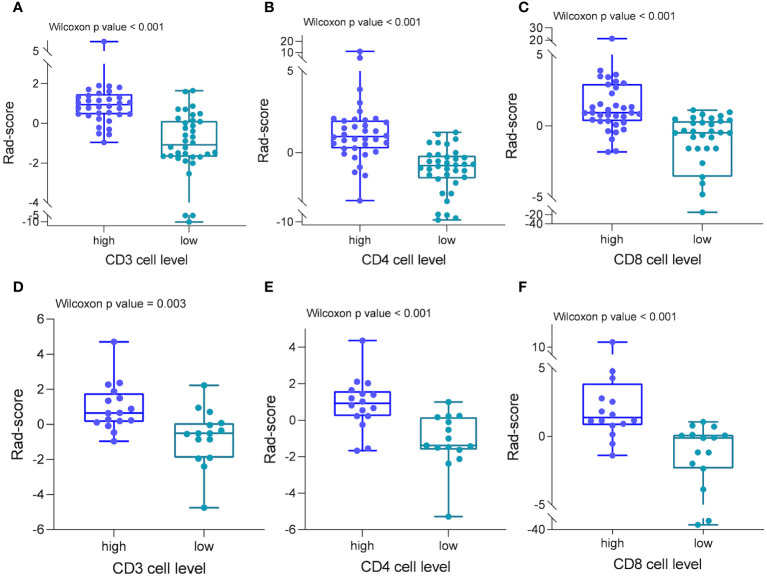
Radiomics scores in different cohorts of patients. In both the training **(A–C)** and test groups **(D–F)**, patients with strong CD3, CD4, and CD8 cell infiltration had significantly higher radiomics scores than patients with low infiltration.

**Figure 5:**
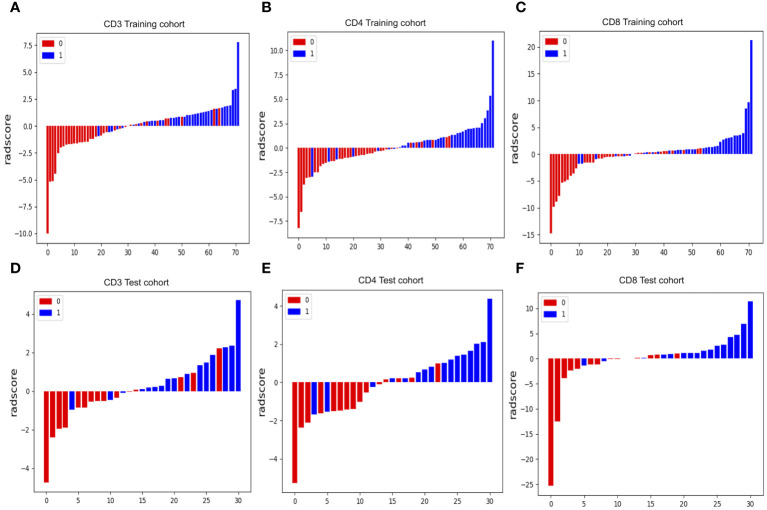
Radiomics score (Rad-score) waterfall plots for CD3 **(A, D)**, CD4 **(B, E)** and CD8 **(C, F)** cohorts. The Y-axis displays Rad-score values. Positive numbers represent high expression forecasts, whereas negative values represent low expression expectations. Correct predictions have red bars with negative values and blue bars with positive values, whereas incorrect predictions have blue bars with negative values and red bars with positive values.


From the DCE-MRI data, 231 features in total were retrieved (67 features each from K^trans^, K_ep_, and V_e_). Details of all texture parameters extracted are provided in the **Supplementary Material**. Then, using the variance thresholding approach (threshold = 0.8), SelectKBest, and LASSO regression algorithms, we screened 8, 8, and 7 variables to build predictive models for CD3, CD4, and CD8, respectively. These attributes were given weights based on the appropriate coefficients. The Rad-score of the high-expression group was greater than that of the low-expression group in both the training and testing datasets of CD3, CD4, and CD8 (P < 0.05) (**Figure 4**). Rad scores for each patient in the training and test sets are presented as bars (**Figure 5**). The Rad-score equation for predicting CD3, CD4, and CD8 was as follows:

$$\begin{array}{c} "\mathrm{Rad}-\mathrm{scoreCD}3=\;0.3027*\mathrm{differenceEntropy}{\text{K}}_{\mathrm{ep}}+\;0.2016*\mathrm{GrayLevelNonuniformity}{\text{K}}_{ep}+\\ 0.5961*\mathrm{SurfaceVolumeRatio}{\text{K}}_{\mathrm{ep}}-\;0.4203*\mathrm{FrequencySize}{\text{V}}_{\text{e}}+\;1.1942*\mathrm{Quantile}5{\text{V}}_{\text{e}}-\\ 0.3383*\mathrm{VoxelValueSum}{\text{K}}_{\mathrm{ep}}-\;0.6013*\mathrm{RelativeDeviation}{\text{K}}_{\text{ep}}-0.1957*\mathrm{GlcmTotalFrequency}{\text{K}}_{\text{ep}}" \end{array}$$

$$\begin{array}{c} "\mathrm{Rad}-\mathrm{scoreCD}4=\;0.0005*\mathrm{differenceVariance}{\text{K}}_{\text{ep}}-\;1.2295*\mathrm{uniformity}{\text{K}}^{\text{trans}}-\\ 0.9835*\mathrm{stdDeviation}{\text{K}}_{\text{ep}}-\;1.5579*\mathrm{Correlation}{\text{K}}_{\text{ep}}-\;0.4702*\mathrm{Inertia}{\text{K}}_{\text{ep}}+\;0.1958*\mathrm{HaraVariance}{\text{K}}_{\text{ep}}\\ +\;1.1830*\mathrm{sumVariance}{\text{K}}_{\text{ep}}+\;0.3908*\mathrm{skewness}{\text{K}}^{\text{trans}}" \end{array}$$

$$\begin{array}{c} "\mathrm{Rad}-\mathrm{scoreCD}8=0.2341*\mathrm{HighGrayLevelRunEmphasis}{\text{K}}_{\text{ep}}+\\ 0.2341*\mathrm{ShortRunHighGrayLevelEmphasis}{\text{K}}_{\text{ep}}+\;0.2341*\mathrm{LongRunHighGrayLevelEmphasis}{\text{K}}_{\text{ep}}-\\ 0.6521*\mathrm{GlcmEnergy}{\text{V}}_{\text{e}}-\;0.7020*\mathrm{InverseDifferenceMoment}{\text{V}}_{\text{e}}-\;4.0937*\mathrm{ClusterShade}{\text{K}}_{\text{ep}}+\\ 0.6728*\mathrm{HaralickCorrelation}{\text{K}}_{\text{ep}}" \end{array}$$

### 3.3 Radiomics model development and evaluation

**Figure 6:**
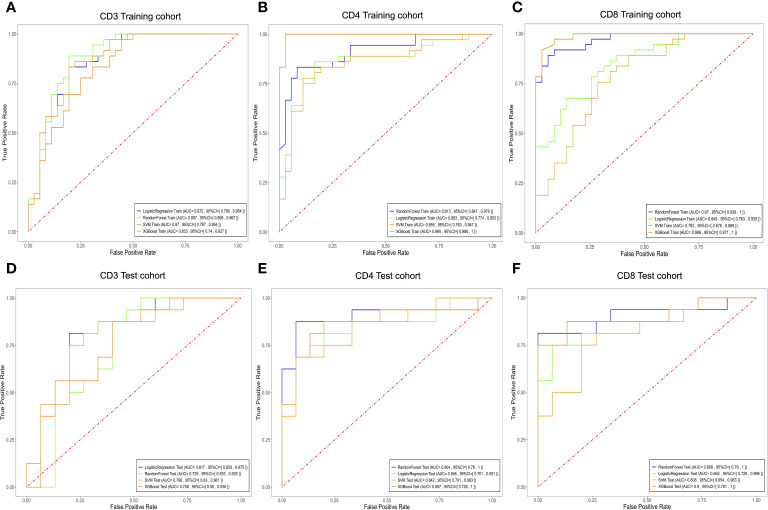
Evaluate the efficacy of different T cell expressions using the LR, RF, XGBoost, and SVM models. Receiver operating characteristic curves for biomarkers used to classify CD3 **(A, D)**, CD4 **(B, E)**, and CD8 **(C, F)** expression levels in the training and testing cohorts. LR, Logistic Regression; SVM, Support Vector Machine; RF, RandomForest; XGBoost, eXtreme Gradient Boosting.

For the prediction models of CD3^+^, CD4^+^, and CD8^+^ T lymphocytes, we constructed and evaluated models using LR, RF, SVM, and XGBoost classifiers. The performance of the classifiers is presented in **Table 4** and **Figure 6**. In the training set, the LR model performed best in predicting CD3 T cells, with high accuracy, sensitivity, specificity, and AUC. In the test set, the LR model for CD3 T cells showed an accuracy of 0.807, sensitivity of 0.813, specificity of 0.800, and AUC of 0.817 (**Figures 6A, D**; **Table 4**). For CD4^+^ and CD8 T^+^ cells, the XGBoost model performed best in the training set, but the RF model showed superior performance in the test set, with higher accuracy and specificity. Specifically, the RF model for CD4^+^ T cells achieved an accuracy of 0.903, sensitivity of 0.875, specificity of 0.933, and AUC of 0.904 in the test set (**Figures 6B, E**; **Table 4**); while for CD8^+^ T cells, the RF model achieved an accuracy of 0.903, sensitivity of 0.813, specificity of 1.000, and AUC of 0.908 in the test set (**Figures 6C, F**; **Table 4**). Therefore, we selected the RF model as the best predictive model for CD4^+^ and CD8^+^. These results indicate that the RF model performs well in predicting CD4^+^ and CD8^+^ T cells, while the LR model exhibits better performance in predicting CD3^+^ T cells. It is worth noting that the XGBoost model may suffer from overfitting, thus we chose random forest as the final predictive model. These findings demonstrate the potential of the developed models for the preoperative prediction of CD3, CD4, and CD8 expression levels in AGC patients.

**Table 4:** The performance of the radiomics model using LR, RF, XGBoost, and SVM classifiers for predicting the extent of CD3, CD4, and CD8 infiltration in each cohort.

	Classifier	Cohort	AUC	ACC	SEN	SPE	PPV	NPV
**CD3**	LR	Training	0.872	0.819	0.833	0.806	0.811	0.829
		Test	0.817	0.807	0.813	0.800	0.813	0.800
	RF	Training	0.887	0.847	0.889	0.806	0.821	0.879
		Test	0.729	0.742	0.875	0.600	0.700	0.818
	SVM	Training	0.870	0.819	0.833	0.806	0.811	0.829
		Test	0.796	0.774	0.750	0.800	0.800	0.750
	XGBoost	Training	0.833	0.764	0.778	0.75	0.757	0.771
		Test	0.758	0.742	0.875	0.600	0.700	0.818
**CD4**	LR	Training	0.863	0.847	0.861	0.833	0.838	0.857
		Test	0.846	0.807	0.688	0.933	0.917	0.737
	RF	Training	0.913	0.875	0.833	0.917	0.909	0.846
		Test	0.904	0.903	0.875	0.933	0.933	0.875
	SVM	Training	0.855	0.833	0.778	0.889	0.875	0.800
		Test	0.842	0.807	0.688	0.933	0.917	0.737
	XGBoost	Training	0.995	0.986	1.000	0.972	0.973	1.000
		Test	0.867	0.807	0.813	0.800	0.813	0.800
**CD8**	LR	Training	0.849	0.764	0.676	0.857	0.833	0.714
		Test	0.863	0.839	0.750	0.933	0.923	0.778
	RF	Training	0.970	0.917	0.892	0.943	0.943	0.892
		Test	0.908	0.903	0.813	1.000	1.000	0.833
	SVM	Training	0.782	0.736	0.757	0.714	0.737	0.735
		Test	0.808	0.774	0.750	0.800	0.800	0.750
	XGBoost	Training	0.988	0.944	0.919	0.971	0.971	0.919
		Test	0.900	0.871	0.750	1.000	1.000	0.790

LR, Logistic Regression; SVM, Support Vector Machine; RF, RandomForest; XGBoost, eXtreme Gradient Boosting; AUC, area under the curve; ACC, accuracy; SEN, sensitivity; SPE, specificity; PPV, positive predictive value; NPV, negative predictive value.

## 4 Discussion

In this study, a noninvasive DCE-MRI-based radiomics model was established and validated to predict preoperative CD3^+^, CD4^+,^ and CD8^+^ T-cell infiltration status in AGC patients. Our research findings underscore the potential of the DCE-MRI radiomics model in assessing CD3^+^, CD4^+^, and CD8^+^ T lymphocyte infiltration levels. This noninvasive assessment method holds significant implications, as it has the potential to assist clinical practitioners in identifying AGC patients who may benefit from immunotherapy, thus providing support for the development of personalized treatment strategies.

Specific biomarkers connected to prognosis and responses to chemotherapy and immunotherapy have been discovered using TME quantitative analysis of diverse cellular subpopulations (11, 20). Previous research in GC has indicated that larger numbers of CD3^+^, CD4^+^, and CD8^+^ T cells within tumors are related to increased overall survival (21, 22). These proteins are normally detected using samples obtained through biopsy or surgical resection, followed by immunohistochemistry examination. However, these analyses can only reflect a part of the tumor tissue and cannot account for the tumor’s overall heterogeneity (23). Imaging, on the other hand, can offer a comprehensive assessment of the overall anatomical structure and functional properties of tumor tissue (24). Much earlier research has shown that radiomics may accurately predict the immune microenvironment in a variety of malignancies using various imaging modalities (25, 26). DCE-MRI technology was used in our study to build a predictive model. This approach varies from traditional MRI imaging in that it offers precise information about the tumor’s structure and function, such as blood volume, vascular permeability, and the vascular network within the tumor (27). This detailed structural and functional investigation aids us in better understanding tumor biology. Previous research has demonstrated that DCE-MRI is capable of predicting the presence of tumor-infiltrating lymphocytes in malignant tumors (28, 29). However, no study has focused on determining the extent of CD3^+^, CD4^+^, and CD8^+^ T-cell infiltration in advanced gastric cancer. In this investigation, we created four ML models utilizing DCE-MRI data and assessed and compared their efficacy in quantifying the numbers of tumor-infiltrating T cells, including CD3, CD4, and CD8 subsets, in advanced gastric cancer patients. This research covers a previously unknown knowledge gap in this field.

This study employed a 10-fold cross-validation approach and trained four machine learning models using pharmacokinetic radiomic features extracted from DCE-MRI data. These models performed admirably in differentiating between different levels of CD3, CD4, and CD8 invasion. The performance evaluation of various machine learning classifiers in predicting tumor-infiltrating T cell levels, including CD3, CD4, and CD8 subpopulations, reveals insights into the effectiveness of these models for clinical applications. For CD3 prediction, LR and SVM classifiers demonstrated robust performance in the training cohort, achieving AUC values of 0.872 and 0.870, respectively. However, in the test cohort, LR exhibited superior performance with an AUC of 0.817, indicating its efficacy in predicting CD3^+^ T cell infiltration. Regarding CD4 prediction, the RF classifier emerged as the top performer with AUC values of 0.913 and 0.904 in the training and test cohorts, respectively. This highlights the capability of RF in accurately predicting CD4^+^ T cell infiltration levels in AGC patients. Similarly, for CD8 prediction, the RF classifier demonstrated excellent predictive ability with AUC values of 0.970 and 0.908 in the training and test cohorts, respectively. The RF model’s high accuracy and specificity suggest its suitability for identifying CD8^+^ T cell infiltration in AGC patients. However, it is worth noting that the XGBoost classifier, while achieving competitive AUC values in the training cohorts for CD3, CD4, and CD8 predictions, exhibited lower performance in the test cohorts, indicating potential overfitting issues. Overall, our findings underscore the potential of the RF classifier as the preferred model for predicting T-cell infiltration levels in AGC based on DCE-MRI data. RF is a robust ensemble learning algorithm that leverages multiple decision trees to achieve high accuracy and incorporates feature selection during classification prediction (30). The robust performance of RF highlights its clinical relevance and utility in guiding treatment decisions and patient management strategies. Nevertheless, further validation in larger and more diverse patient cohorts is warranted to confirm the generalizability and reliability of the predictive models in real-world clinical settings.

In our study, K_ep_ features played a pivotal role in constructing our radiomic model. K_ep_ reflects the rate at which the contrast agent returns from the extravascular-extracellular space (EES) to the vasculature, providing crucial insights into tumor vascular characteristics and the distribution of the contrast agent within tissues (31). Typically, K_ep_ values in tumor tissues are higher because the vasculature network in malignant tumors tends to be more tortuous, irregular, and permeable, resulting in rapid ingress and egress of contrast agents within the tissue (32). Previous research has underscored the significance of K_ep_ in predicting the biological characteristics of tumors, including the extent of immune cell infiltration (33). This is because tumor vascular permeability and blood flow are closely associated with immune cell infiltration within tumor tissues. Thus, the prominence of K_ep_ features in our radiomic model is justified, as they furnish a profound understanding of the tumor vascular microenvironment, which is critical for comprehending the distribution and infiltration of immune cells within tumors.

## 5 Limitations

First, we should note that the data for this retrospective analysis come from only one institution, which could contribute to selection bias. Furthermore, because the predictive model has not been externally validated, there is an urgent need to undertake additional prospective research, particularly multicenter trials involving various medical centers. Second, due to the relatively limited sample size, additional patients will need to be recruited in future research to further evaluate the model’s dependability. Finally, we did not directly validate the correlation between immune cell levels and actual immunotherapy outcomes. To comprehensively assess our model’s predictive capability, future research could consider validating these predictions in clinical practice, particularly by incorporating patient groups undergoing immunotherapy. This would help determine whether our model has the potential to serve as a clinical tool to assist in immunotherapy decisions. In particular, we did not include a thorough comparison between existing approaches to T-cell infiltration prediction and our suggested DCE-MRI radiomics model. To properly evaluate our model’s potential in clinical practice, future research might look into examining how our model varies from other approaches in terms of accuracy, dependability, and clinical application. Despite these limitations, radiomics models hold promise for precision and personalized medicine in AGC patients.

## 6 Conclusion

In conclusion, this work demonstrates the utility of DCE-MRI radiomics analysis in distinguishing levels of CD3^+^, CD4^+^, and CD8^+^ T lymphocyte infiltration in pretreatment AGC patients. This discovery highlights magnetic resonance imaging’s potential as a noninvasive diagnostic for predicting the expression of immunotherapy-related proteins.

## Data availability statement

The raw data supporting the conclusions of this article will be made available by the authors, without undue reservation.

## Ethics statement

The requirement of ethical approval was waived by The Ethics Committee of Shaoxing People’s Hospital for the studies involving humans because ethical review and approval was not required for the study on human participants in accordance with the local legislation and institutional requirements. The studies were conducted in accordance with the local legislation and institutional requirements. The ethics committee/institutional review board also waived the requirement of written informed consent for participation from the participants or the participants’ legal guardians/next of kin because written informed consent from the participants was not required to participate in this study in accordance with the national legislation and the institutional requirements. Written informed consent was not obtained from the individual(s) for the publication of any potentially identifiable images or data included in this article because Written informed consent from the participants was not required to participate in this study in accordance with the national legislation and the institutional requirements.

## Author contributions

HH: Data curation, Investigation, Resources, Visualization, Writing – original draft, Writing – review & editing. LZ: Funding acquisition, Investigation, Methodology, Project administration, Resources, Writing – review & editing. DW: Data curation, Funding acquisition, Resources, Validation, Writing – review & editing. YY: Data curation, Resources, Writing – review & editing. HJ: Data curation, Resources, Writing – review & editing. ZL: Funding acquisition, Methodology, Project administration, Supervision, Writing – review & editing.
