# Supplementary material for: Machine learning models based on quantitative dynamic contrast-enhanced MRI parameters assess the expression levels of CD3+, CD4+, and CD8+ tumor-infiltrating lymphocytes in advanced gastric carcinoma
Source: Front Oncol. 2024 Mar 14;14:1365550. doi: 10.3389/fonc.2024.1365550 (PMC10973004; doi:10.3389/fonc.2024.1365550)
Supplement: Supplementary file 1 [file DataSheet_1.docx]

Supplementary Material

Machine learning models based on quantitative dynamic contrast-enhanced MRI parameters assess the expression levels of CD3+, CD4+, and CD8+ tumor-infiltrating lymphocytes in advanced gastric carcinoma

Huizhen Huang *, Zhiheng Li, Dandan Wang, Ye Yang, HongYan Jin

*** Correspondence:** Zengxin Lu: luzx777@163.com

# Supplementary Figures and Tables

## Supplementary Figures


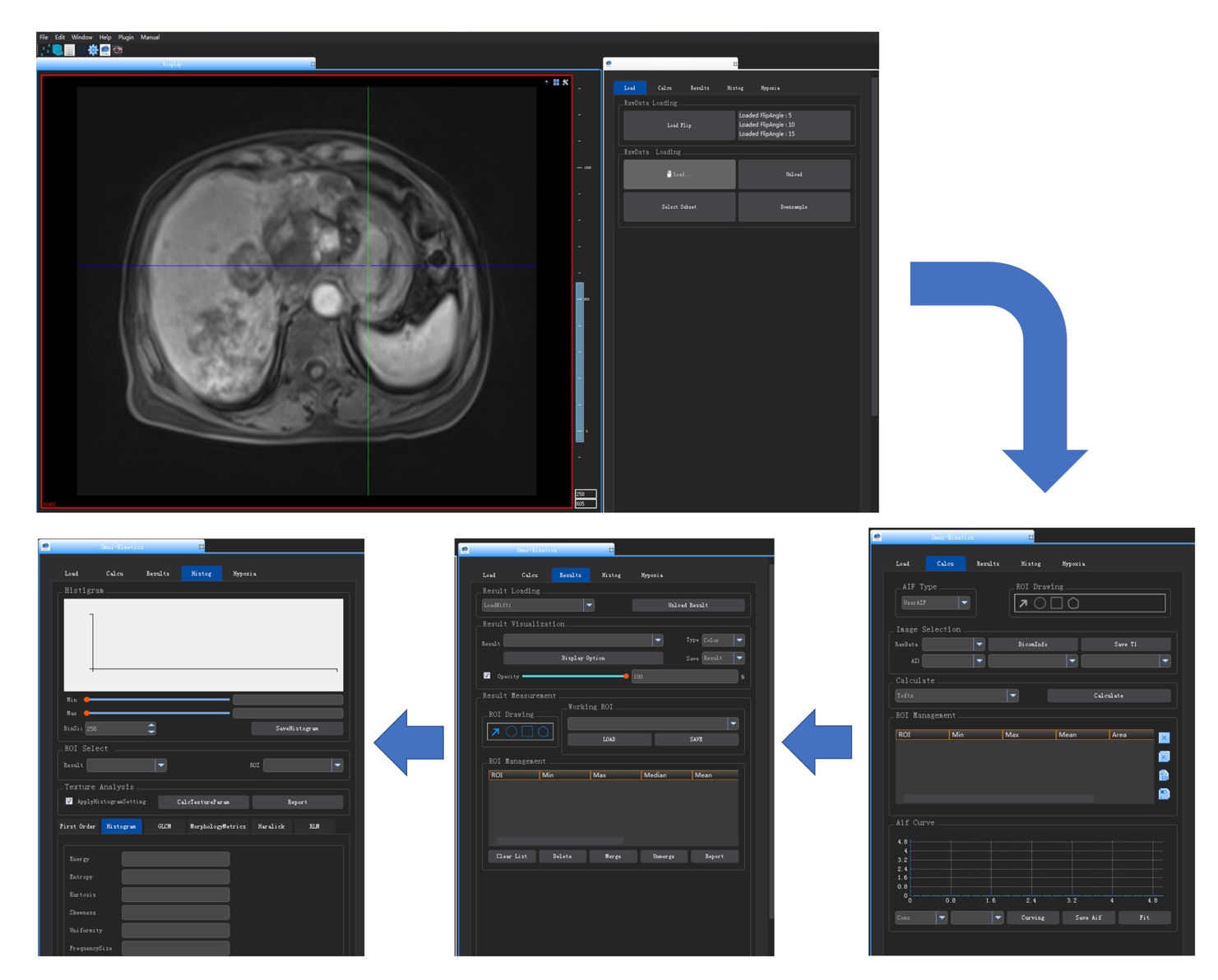


**Supplementary Figure 1.** The interface and process of Omni Kinetics software for DCE-MRI image processing and feature extraction.

## Supplementary Tables

**Supplementary Table 1.** Radiomics features derived from dynamic contrast-enhanced magnetic resonance imaging using OmniKinetics software.

Note: Gray-level Co-occurrence Matrix (GLCM); Run Length Matrix (RLM).

| **Algorithms** | **First Order** | **Histogram** | **GLCM** | **Haralick** | **RLM** |
| --- | --- | --- | --- | --- | --- |
| **Radiomic features** | MinIntensity, MaxIntensity, MedianIntensity, MeanValue, stdDeviation, Variance, VolumeCount, VoxelValueSum, Root Mean Square, Range, MeanDeviation, RelativeDeviation, MinLocation, MaxLocation | Quantile5, Quantile10, Quantile25, Quantile50, Quantile75, Quantile90, Quantile95, Energy, Entropy, Kurtosis, Skewness, Uniformity, FrequencySize, Uniformity Positive Pixel, Mean Positive Pixel | GlcmEnergy, GlcmEntropy, GlcmBinSize, GlcmTotalFrequency, GlcmMatrixMean, GlcmRelativeFrequency, Inertia, Correlation, InverseDifferenceMoment, ClusterShade, ClusterProminence, HaralickCorrelation, InvalidFeatureName | AngularSecondMoment, Contrast, HaraVariance, sumAverage, sumVariance, sumEntropy, differenceVariance, differenceEntropy, inverseDifferenceMoment | MaxIntensity, MinIntensity, MinSize, NumberOfIntensityBins, MaxSize, NumberOfSizeBins, ShortRunEmphasis, LongRunEmphasis, GreyLevelNonuniformity, RunLengthNonuniformity, LowGreyLevelRunEmphasis, HighGreyLevelRunEmphasis, ShortRunLowGreyLevelEmphasis, ShortRunHighGreyLevelEmphasis, LongRunLowGreyLevelEmphasis, LongRunHighGreyLevelEmphasi |
